# Neoadjuvant chemotherapy using platinum- and taxane-based regimens for bulky stage Ib2 to IIb non-squamous cell carcinoma of the uterine cervix

**DOI:** 10.1007/s00280-012-2052-2

**Published:** 2012-12-23

**Authors:** Tadahiro Shoji, Eriko Takatori, Tatsunori Saito, Hideo Omi, Masahiro Kagabu, Fumiharu Miura, Satoshi Takeuchi, Toru Sugiyama

**Affiliations:** Department of Obstetrics and Gynecology, Iwate Medical University School of Medicine, 19-1 Uchimaru, Morioka, 020-8505 Japan

**Keywords:** Cervical cancer, Non-squamous cell carcinoma, Neoadjuvant chemotherapy, Paclitaxel, Docetaxel, Carboplatin

## Abstract

**Purpose:**

There are no reports on the use of neoadjuvant chemotherapy (NAC) in non-squamous cell cervical carcinoma. We examined the effectiveness and safety of paclitaxel/carboplatin (TC) and docetaxel/carboplatin (DC).

**Methods:**

Stage Ib2 to IIb disease was present in 23 patients scheduled for radical hysterectomy. We administered 1–3 courses of either the TC or the DC regimen. Anti-tumor effects were found superior by Response Evaluation Criteria in Solid Tumors. Safety was assessed with National Cancer Institute Common Terminology Criteria for Adverse Events.

**Results:**

Median age was 50 years (range 32–63 years), with stage Ib2 in 6 cases (26.1 %) and IIb in 17 cases (73.9 %). Complete response was achieved in 5 cases (21.7 %), partial response in 13 (56.5 %), stable disease in 5 (21.7 %); the response rate was 78.3 %, and surgery completion rate was 78.3 %. Leukopenia or neutropenia ≥grade 3 was seen in 12 (52.2 %) and 21 (91.3 %) cases, respectively, with grade 3 febrile neutropenia in 2 cases (8.7 %) and no anemia or thrombocytopenia ≥grade 3. Median progression-free survival was 26 months (95 % Cl, 13.5–38.5 months); median overall survival was 35 months (95 % Cl, 20.9–49.1 months).

**Conclusion:**

NAC for non-squamous cell cervical carcinoma showed potent anti-tumor effects and manageable adverse events.

## Introduction

The methods used to treat bulky stage Ib2 to IIb cervical cancers differ between Japan and Western countries. In Western countries, concurrent chemoradiation therapy (CCRT) has been recommended as a standard treatment for such tumors, based on the results of multiple large-scale randomized trials and meta-analyses [1–7]. In Japan, Korea, and Italy, among other countries, the neoadjuvant chemotherapy (NAC) approach has been introduced to clinical practice and is extensively utilized [8, 9]. An Italian phase III, controlled study involving patients with locally advanced stage Ib2 to IIb squamous cell carcinoma of the cervix showed that NAC prior to radical hysterectomy improves patient outcomes compared with conventional radiation therapy alone [10].

There are no previous reports on the use of NAC for bulky non-squamous cell carcinoma of the cervix. We present the results of an ongoing pilot study on its efficacy and safety.

## Subjects and methods

### Subjects

We studied 23 patients with locally advanced non-squamous cell carcinoma of the uterine cervix (clinical stage Ib2 to IIb) between January 2002 and September 2011. All patients were scheduled to undergo radical hysterectomy and gave informed consent for this study.

### Inclusion criteria

The following inclusion criteria were employed: (1) histologically verified non-squamous cell carcinoma of the uterine cervix; (2) locally advanced disease, stage Ib2–IIb; (3) between 20 and 74 years of age; (4) Eastern Cooperative Oncology Group performance status 0–2; (5) no prior treatment; (6) presence of a measurable bulky mass in the uterine cervix on magnetic resonance imaging (MRI); (7) hematologic and biochemical findings within the following parameters, WBC count ≥4,000/mm^3^, neutrophil count ≥2,000/mm^3^, platelet count ≥100,000/mm^3^, hemoglobin ≥10.0 g/dL, AST and ALT levels ≤2 times the upper limit of normal reference range, serum total bilirubin level ≤1.5 mg/dL, serum creatinine ≤1.5 mg/dL, and creatinine clearance ≥60 mL/min; (8) life expectancy ≥6 months; and (9) written informed consent personally given by the subject.

### Exclusion criteria

Exclusion criteria were as follows: (1) overt infection; (2) serious complication(s), for example, cardiac disease, poorly controlled diabetes mellitus, malignant hypertension, bleeding tendency; (3) multiple active cancers; (4) interstitial pneumonia or pulmonary fibrosis; (5) pulmonary effusions; (6) history of unstable angina or myocardial infarction within 6 months after registration, or a concurrent serious cardiac arrhythmia requiring treatment; (7) contraindications to treatment with paclitaxel, docetaxel, or carboplatin; (8) intestinal paralysis or ileus; (9) pregnancy, breast-feeding, or desire for future pregnancy; (10) history of serious drug hypersensitivity or drug allergy; and (11) judged unsafe for participation by the attending physician.

### Medication administration and criteria for modification

#### Regimen

The choice of regimen was left to the attending physician. Paclitaxel/carboplatin (TC) therapy was administered to 4 patients and DC therapy to 19 patients. Courses of treatment were administered 21 days apart, with a intravenous paclitaxel dose of 175 mg/m^2^ or a docetaxel dose of 70 mg/m^2^ administered on Day 1, and intravenous carboplatin with area under the curve (AUC) 6 mg/mL per min also administered on Day 1. As a rule, maximum 3 courses of treatment were administered to each patient.

#### Criteria for initiating the second course of treatment

The second course was postponed by a maximum of 2 weeks when blood analysis performed within 2 days prior to the planned start did not satisfy the following criteria: (1) neutrophil count ≥1,000/mm^3^; (2) platelet count ≥75,000/mm^3^.

#### Carboplatin dose-reduction criteria

The carboplatin dose for the second course was reduced from AUC 6 mg/mL per min to AUC 5 mg/mL per min if the patient experienced grade 4 thrombocytopenia or grade 3 thrombocytopenia accompanied by bleeding. If signs of toxicity remained after this dose reduction, the third course of treatment was reduced to AUC 4.

#### Paclitaxel dose-reduction criteria

The paclitaxel dose for the second course was reduced from 175 to 135 mg/m^2^ in patients exhibiting grade 2 or higher severe peripheral nerve toxicity during the first course. If this grade of nerve toxicity persisted after the dose reduction, the paclitaxel dose for the third course was reduced to 110 mg/m^2^.

#### Docetaxel dose-reduction criteria

The docetaxel dose for the second course was reduced from 70 to 60 mg/m^2^ if the patient experienced grade 4 neutropenia lasting 7 days or longer or febrile neutropenia lasting 4 days or longer. If signs of toxicity remained after this dose reduction, the docetaxel dose for the third course was reduced to 50 mg/m^2^.

### Supportive therapy

A granulocyte colony-stimulating factor (G-CSF) preparation was administered to patients who developed grade 4 neutropenia during the first course of NAC. These patients were permitted prophylactic G-CSF during the second and subsequent courses of NAC. Anti-emetics were used for the preventive purpose.

### Primary treatment

Patients with stage Ib2–IIb cervical carcinoma underwent radical hysterectomy unless the tumor responded to preoperative treatment with progressive disease (PD), at which time the tumor was up-staged. In cases in which surgery was not possible, CCRT was adopted.

### Postoperative therapy

Postoperative radiotherapy, postoperative chemotherapy, or CCRT was additionally administered in patients with a positive surgical margin at the vaginal stump, lymphadenopathy, invasion of the cardinal ligament, or evident invasion of the vasculature.

### Outcome evaluation

The primary endpoint was anti-tumor response. Secondary endpoints comprised adverse events, the surgery completion rate, the progression-free survival (PFS) period, and the overall survival (OS) period. Hematologic tests and urinalysis were performed before the start of treatment and, as a rule, once weekly after starting treatment. Electrocardiograms and chest radiographs were obtained before the start and at the end of treatment.

#### Evaluation of anti-tumor response

Anti-tumor response was evaluated using Response Evaluation Criteria in Solid Tumors guidelines. The baseline MRI findings were compared with the findings at the conclusion of treatment. For our efficacy evaluation, we adopted the best rating, without incorporating the response period.

#### Evaluation of adverse events

Adverse events were evaluated employing the National Cancer Institute Common Terminology Criteria for Adverse Events, version 3.0.

### Statistical analysis

Progression-free survival (PFS), defined as the time from the start of the study treatment to documented tumor progression or death, and overall survival (OS), defined as the time from the start of treatment to the date of death, were calculated by the Kaplan–Meier method. The statistical data were obtained using StatMate III.

## Results

### Background variables

The median age of the 23 patients was 50 years (range 32–63 years). The performance status was 0 in 18 patients (78.3 %), and in 5 patients, the performance status was 1 (21.7 %). The clinical stage was Ib2 in 6 cases (26.1 %) and IIb in 17 cases (73.9 %). The histological type was mucinous adenocarcinoma in 10 cases (43.5 %), endometrioid adenocarcinoma in 5 (21.7 %), clear cell adenocarcinoma in 1 (4.3 %), and adenosquamous carcinoma in 7 (30.4 %). One course of NAC was administered in 1 patient (4.3 %), 2 courses in 17 patients (73.9 %), and 3 courses in 5 patients (21.7 %). The regimens comprised TC therapy in 4 cases (17.4 %) and DC therapy in 19 cases (82.6 %) (Table 1).

**Table 1:** Patient characteristics (*n* = 23)

Median age years [range]		Cell type	
50 [32–63]		Mucinous	10 (43.5 %)
Performance status at entry		Endometrioid	5 (21.7 %)
0	18 (78.3 %)	Clear cell	1 (4.3 %)
1	5 (21.7 %)	Adenosquamous	7 (30.4 %)
2	0 (0 %)	Regimen	
FIGO stage at initial diagnosis		DC	19 (82.6 %)
Ib2	6 (26.1 %)	TC	4 (17.4 %)
IIa	0 (0 %)	Number of cycles	
IIb	17 (73.9 %)	1	1 (4.3 %)
		2	17 (73.9 %)
		3	5 (21.7 %)

*DC* docetaxel + carboplatin, *TC* paclitaxel + carboplatin

### Anti-tumor response

A complete response was noted in 5 cases (21.7 %), partial response in 13 (56.5 %), and stable disease in 5 (21.7 %), with a overall response rate of 78.3 %. In subgroup analysis, the overall response rate of TC therapy and DC therapy was 100 and 73.7 %, respectively (Table 2).

**Table 2:** Response

	CR	PR	SD	PD	Overall response
Total	5	13	5	0	18 (78.3 %)
TC	1	3	0	0	4 (100 %)
DC	4	10	5	0	14 (73.7 %)

*CR* complete response, *PR* partial response, *SD* stable disease, *PD* progressive disease, *TC* paclitaxel + carboplatin, *DC* docetaxel + carboplatin

### Adverse events

Grade 3 or higher severe leukopenia or neutropenia was seen in 12 (52.2 %) and 21 (91.3 %) cases, respectively. Grade 3 febrile neutropenia was noted in 2 cases (8.7 %). The G-CSF preparation was used in 13 (56.5 %) of the 23 patients; it was administered during 19 (38.8 %) of the 49 total cycles. The mean duration of G-CSF treatment during each course was 2.6 days. No patients experienced grade 3 or higher severe anemia or thrombocytopenia. The only sign of grade 3 or higher severe non-hematologic toxicity was nausea, seen in 1 case (4.3 %). No patients had signs of grade 2 or higher severe neurotoxicity (Table 3).

**Table 3:** Adverse events of TC/DC therapy

*n* = 23	Grade				
	1	2	3	4	≧3 (%)
Leukopenia	2	9	11	1	12 (52.2)
Neutropenia	1	1	7	14	21 (91.3)
Thrombocytopenia	11	0	0	0	0
Anemia	11	12	0	0	0
Nausea	11	3	1	0	1 (4.3)
Vomiting	5	3	0	0	0
Diarrhea	2	0	0	0	0
Neurotoxicity	18	0	0	0	0
Dyspnea	3	0	0	0	0
Fibrile neutropenia	0	0	2	0	2 (8.7)

*TC* paclitaxel + carboplatin, *DC* docetaxel + carboplatin

In 3 cases (13.0 %), the second course of treatment was postponed due to a low neutrophil count; in all 3 patients, the second course was initiated within 7 days of its scheduled time. Both patients (10.0 %) with grade 3 febrile neutropenia for 4 days or longer had received DC therapy prior to the development of this complication. In these 2 cases, doses were reduced for the second course of treatment: docetaxel from 70 to 60 mg/m^2^ and carboplatin from AUC 6 to AUC 5.

### Surgery completion and adjuvant therapy

Radical hysterectomy after NAC was completed in 18 of the 23 patients, giving a surgery completion rate of 78.3 %. Adjuvant therapy after radical hysterectomy consisted of no treatment in 3 cases (13.0 %), radiotherapy in 2 cases (8.7 %), chemotherapy in 15 cases (65.2 %), and CCRT in 3 cases (13.0 %).

### Survival

**Fig. 1:**
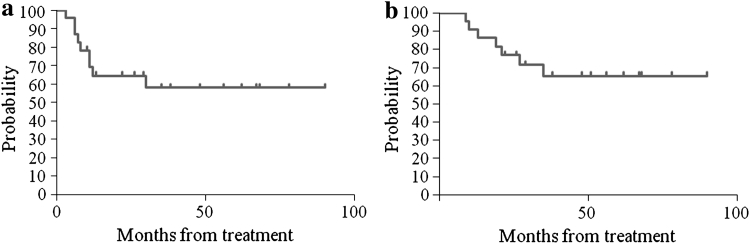
Kaplan–Meier curves for progression-free survival (**a**) and overall survival (**b**). The median PFS for all patients was 26 months (95 % CI, 13.5–38.5 months), and the median OS was 35 months (95 % CI, 20.9–49.1 months)

The median follow-up period was 31 months (range 9–90 months). The median progression-free survival period was 26 months (95 % Cl, 13.5–38.5 months), and the median overall survival period was 35 months (95 % Cl, 20.9–49.1 months) (Fig. 1). The 5 patients in whom surgery was not complete died of their primary disease within 35 months. Their median PFS and OS were 8 months (3–12 months) and 21 months (10–35 months), respectively.

## Discussion

The incidence of non-squamous cell carcinoma of the uterine cervix has been steadily rising in Japan, currently accounting for approximately 10–15 % of all cervical cancer cases. Lymph node metastasis is more frequent with this disease, compared with invasive squamous cell carcinoma [11], and its sensitivity to radiotherapy and chemotherapy is considered to be lower [12]. Thus, squamous and non-squamous cell carcinomas must be analyzed separately. It is advisable and desirable to try new therapeutic strategies in non-squamous cell carcinoma, but the number of published studies involving this type of cervical cancer is small, with the number of cases analyzed in these reports also small. Thus, no high-level evidence regarding treatment has been obtained for this type of cervical carcinoma.

The response rates of adenocarcinoma are reportedly 20 % to cisplatin [13], 15 % to ifosfamide [14], 14 % to 5-fluorouracil [15], and 12 % to oral etoposide [16]; these response rates are lower than those of squamous cell carcinoma. According to Curtin et al. [17], however, the response rate of adenocarcinoma to paclitaxel is as high as 31 %, even when the agent is used independently. Docetaxel has also been attracting interest as an agent of NAC. Nagao et al. evaluated the efficacy of combined chemotherapy using a DC regimen (docetaxel 60 mg/m^2^ and carboplatin at AUC 6 on day 1, repeating the combination every 21 days) in 17 patients with advanced or recurrent cervical cancer, including 6 with adenocarcinoma and 1 with adenosquamous carcinoma. A partial response was obtained in 6 of the 7 cases with adenocarcinoma (including the case of adenosquamous carcinoma); the response rate was 86 % [18]. Considering these findings, we conducted a pilot study involving standard regimens of TC and DC, conventionally used for the treatment of ovarian cancer.

In the analysis of adverse events, severe neutropenia developed in 91.3 % of patients, but subsided in response to short-term treatment with a G-CSF preparation. During the first course of DC therapy, grade 3 febrile neutropenia developed in 2 cases; the dose of both agents was reduced for the next course of treatment. All signs, specific to taxanes, of peripheral neuropathy were grade 1 or less, allowing for continuation of treatment while preserving the quality of life of the individual patients. No serious adverse events occurred, and the response rate was 78.3 %. This study demonstrated a high response rate of bulky non-squamous cell carcinoma of the cervix to NAC using taxanes (paclitaxel or docetaxel) and carboplatin. It also demonstrated the safety of the medications in this regimen. The completion rate of radical hysterectomy, however, was only 78.3 %; thus, the treatment outcomes in this study were not satisfactory. Possible reasons for the low surgery completion rate include the rapid progression of non-squamous cell carcinoma, frequent invasion of tissues and organs surrounding the uterus, and frequent lymph node metastasis.

The treatment results and outcomes of all patients were shown in Table 4. Unfortunately, all patients with incomplete surgery ultimately experienced disease recurrence and died of their primary disease. Thus, the significance of NAC at present may not be to prolong survival time. Instead, in our view, NAC should be performed to fully optimize patients’ conditions with its antitumor effect in order to improve the chances of complete surgery. Further study is needed regarding the long-term outcomes of NAC.

**Table 4:** Treatment results and outcomes of all patients

Patients	Age	Stage	Cell type	Regimen	Cycles	Responses	Surgery	Adjuvant	Follow-up period (months)	PFS (months)	OS (months)	Outcome
1	52	IIb	ASC	DC	2	CR	Incomplete	CCRT	21	12	21	DOD
2	50	Ib2	MAC	TC	2	CR	Complete	NT	90	90	90	NED
3	55	IIb	ASC	DC	2	CR	Complete	CT	62	62	62	NED
4	39	Ib2	MAC	DC	3	CR	Complete	CT	51	11	51	AWD
5	36	Ib2	MAC	DC	2	CR	Complete	CT	22	22	22	NED
6	32	IIb	ASC	DC	3	PR	Incomplete	NT	19	11	19	DOD
7	49	Ib2	MAC	DC	2	PR	Complete	CCRT	78	78	78	NED
8	60	IIb	ASC	DC	2	PR	Complete	NT	68	30	68	AWD
9	54	IIb	EDC	TC	1	PR	Complete	CT	68	68	68	NED
10	40	Ib2	MAC	TC	2	PR	Complete	CT	67	67	67	NED
11	38	IIb	MAC	DC	2	PR	Complete	CT	9	6	9	DOD
12	63	IIb	CCC	DC	2	PR	Complete	RT	48	48	48	NED
13	50	IIb	EDC	DC	2	PR	Complete	CT	35	35	35	NED
14	53	Ib2	EDC	DC	2	PR	Complete	CT	38	38	38	NED
15	54	IIb	MAC	DC	2	PR	Complete	CT	29	29	29	NED
16	52	IIb	MAC	TC	3	PR	Incomplete	CT	27	7	27	DOD
17	45	IIb	EDC	DC	2	PR	Complete	CT	10	10	10	NED
18	51	IIb	EDC	DC	3	PR	Complete	CT	13	13	13	NED
19	45	IIb	MAC	DC	2	SD	Incomplete	CT	10	3	10	DOD
20	52	IIb	ASC	DC	2	SD	Complete	CT	13	6	13	DOD
21	56	IIb	ASC	DC	2	SD	Complete	CCRT	56	56	56	NED
22	61	IIb	ASC	DC	3	SD	Incomplete	RT	35	8	35	DOD
23	45	IIb	MAC	DC	2	SD	Complete	CT	26	26	26	NED

*ASC* adenosquamous cell carcinoma, *MAC* mucinous adenocarcinoma, *EDC* endometrioid adenocarcinoma, *CCC* clear cell adenocarcinoma, DC docetaxel + carboplatin, *TC* paclitaxel + carboplatin, *CR* complete response, *PR* partial response, *SD* stable disease, *NT* no treatment, *CT* chemotherapy, *RT* radiotherapy, *CCRT* concurrent chemoradiation therapy, *PFS* progression-free survival, *OS* overall survival, *NED* no evidence of disease, *AWD* alive with disease, *DOD* died of disease
